# Emerging roles of microRNAs in septic cardiomyopathy

**DOI:** 10.3389/fphar.2023.1181372

**Published:** 2023-07-05

**Authors:** Mingyan Wu, Guangdong Li, Wenjun Wang, Hongsheng Ren

**Affiliations:** Department of Intensive Care Unit, Shandong Provincial Hospital Affiliated to Shandong First Medical University, Jinan, Shandong, China

**Keywords:** septic cardiomyopathy, microRNA, autophage, inflammation, apoptosis, pyroptosis

## Abstract

As one of the serious complications of sepsis, septic cardiomyopathy has gained more and more attention, because of its high morbidity and mortality. With the in-depth study of septic cardiomyopathy, several methods have been adopted clinically but have poor therapeutic effects due to failure to find precise therapeutic targets. In recent years, microRNAs have been found to be related to the pathogenesis, diagnosis, and treatment of septic cardiomyopathy via regulating immunity and programmed cell death. This paper reviews the role of microRNAs in septic cardiomyopathy, aiming to provide new targets for the diagnosis and treatment of septic cardiomyopathy.

## 1 Introduction

Sepsis, a multiple organ dysfunction disease caused by infection or injury, is regarded as the leading cause of death of patients in the intensive care unit. A study about the incidence and mortality of sepsis showed that in 2017 there were approximately 48.9 million patients with sepsis. And more than 11 million patients die of it, accounting for 19.7% of global deaths (Rudd et al., 2020). Septic cardiomyopathy (SCM) is recognized as acute cardiac dysfunction without effective treatments in more than half of septic patients contributing to increased mortality and morbidity. The morbidity of SCM in patients with sepsis is approximately 30%–50%, with a 28-day case fatality rate of 30% (Beesley et al., 2018). MicroRNAs (miRNAs) are a class of non-coding RNA genes whose mature products are approximately 22-base functional RNA molecules (Griffiths-Jones et al., 2006). In recent years, miRNAs have been found to play an important role in the pathophysiological processes of various diseases, including SCM. Recently, more and more papers have focused on the relationship between miRNAs and SCM. In this paper, for the first time, we review the mechanisms of miRNAs in regulating SCM via immunity and programmed cell death, and the potential role of miRNAs in the precise prevention and treatment of SCM.

## 2 Methods

**FIGURE 1:**
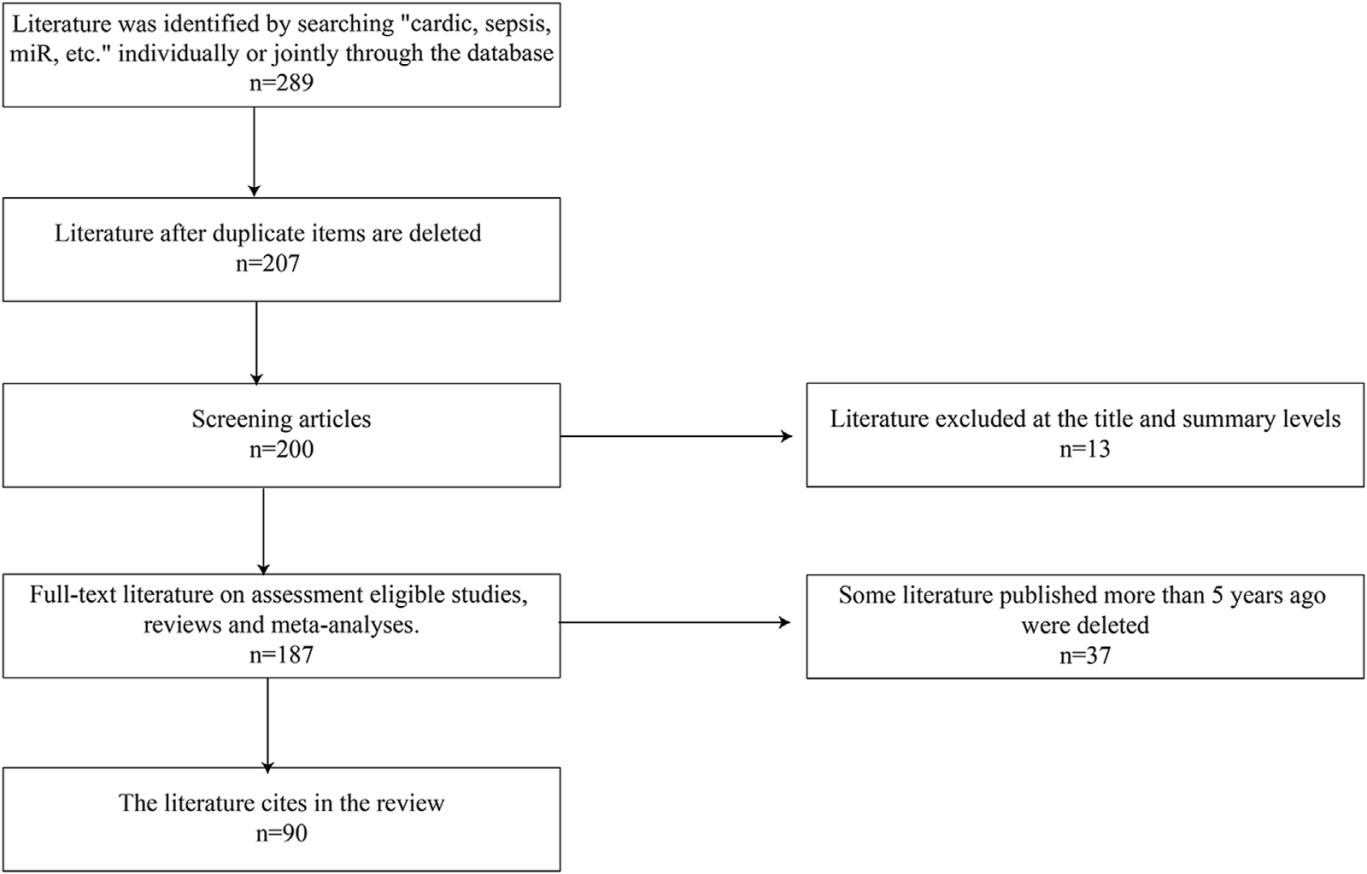
Steps of how the papers are selected.

As described in Figure 1, the literature search was performed using search terms “sepsis,” “sepsis induced cardiomyopathy,” “miRNAs,” “programmed cell death,” “apoptosis,” “immunity,” “autophagy,” and “pyroptosis,” alone or in combination. The article has included both clinical and animal studies focusing on basic mechanisms, pathophysiology, potential clinical applications. All articles included were from peer-reviewed journal in English.

## 3 Sepsis and SCM

### 3.1 Sepsis

The release of multiple inflammatory mediators in patients with sepsis leads to systemic multi-organ dysfunction, such as acute respiratory distress syndrome (ARDS) (Xu et al., 2021), sepsis-associated acute kidney injury (AKI) (Peerapornratana et al., 2019), SCM, disseminated intravascular coagulation (DIC) (Iba et al., 2020)and so on (Font et al., 2020). Since 2004, “severe sepsis bundle” as the treatment of sepsis, has become more acceptable. The latest 2021 guidelines for the treatment of sepsis emphasized the importance of early identification and effective fluid resuscitation (Evans et al., 2021). This guideline touched on a large study proving lower mortality by starting the sepsis bundles successfully, which included 3-hour and 6-hour sepsis bundle (Levy et al., 2018). Septic shock is considered as a subset of sepsis with a higher risk of death than sepsis. Septic shock can be identified as using the clinical criteria of hypotension requiring vasopressor therapy to maintain a mean BP of 65 mmHg or greater and having a serum lactate level greater than 2 mmol/L after adequate fluid resuscitation (Shankar-Hari et al., 2016).

A meta-analysis showed that over 30 million people worldwide are diagnosed with sepsis each year, with a 30-day mortality rate of approximately 24.4%, and a 30-day septic shock mortality rate of 34.7% (Bauer et al., 2020). Mortality rates did not continue to decline under “bundle” therapy, indicating that the need for sepsis treatment remains unsolved. Early recognition of sepsis and prevention of sepsis complications or septic shock are important to reduce the mortality of sepsis and improve the prognosis (Gavelli et al., 2021).

### 3.2 SCM

SCM was initially regarded as a reduction in cardiac ejection fraction in the early stages of septic shock, characterized by left ventricular dilatation and cardiac index decrease (Parker et al., 1984). Some studies have shown that the incidence of SCM is about 40%, and its occurrence increases the sepsis mortality rate from 30% to 70% (Romero-Bermejo et al., 2011). The pathogenesis of SCM is caused by a dysregulated response to inflammation, and the most recognized mechanisms include unbalanced immunity and dysregulated programmed cell death. As same as sepsis, infection control, and appropriate fluid resuscitation remain the first chosen for the treatment of SCM. While β-blockers can reduce the heart rate and myocardial oxygen consumption, dobutamine and norepinephrine are used as vasoactive or inotropic agents (Rhodes et al., 2017). Recent studies have shown that levosimendan can inhibit inflammation and exert a protective effect on cardiac function. However, levosimendan has also been reported to cause arrhythmias as a calcium sensitizer, with no significant effect on mortality (Yamashita et al., 2018). A meta-analysis showed that venoarterial extracorporeal membrane oxygenation (VA-ECMO) in SCM patients may reduce mortality, but currently, this treatment is only applicable to patients with significantly reduced left ventricular ejection fraction (Ling et al., 2021). It is clear that these nonspecific treatments cause limited improvement in the prognosis of SCM, therefore searching for new treatment strategies and therapeutic targets are expected. With the discovery of proteomics and genomics, which have been shown to play a significant role in human disease prevention, differences in the expression of these genes occupy an important place in the pathophysiology of sepsis (Maslove and Wong, 2014). People began to pay attention to the clinical application of miRNAs in the pathogenesis, diagnosis, and prognosis of SCM. Further studies are needed to discover the important role of miRNAs in SCM.

## 4 miRNAs and SCM

### 4.1 miRNAs

**FIGURE 2:**
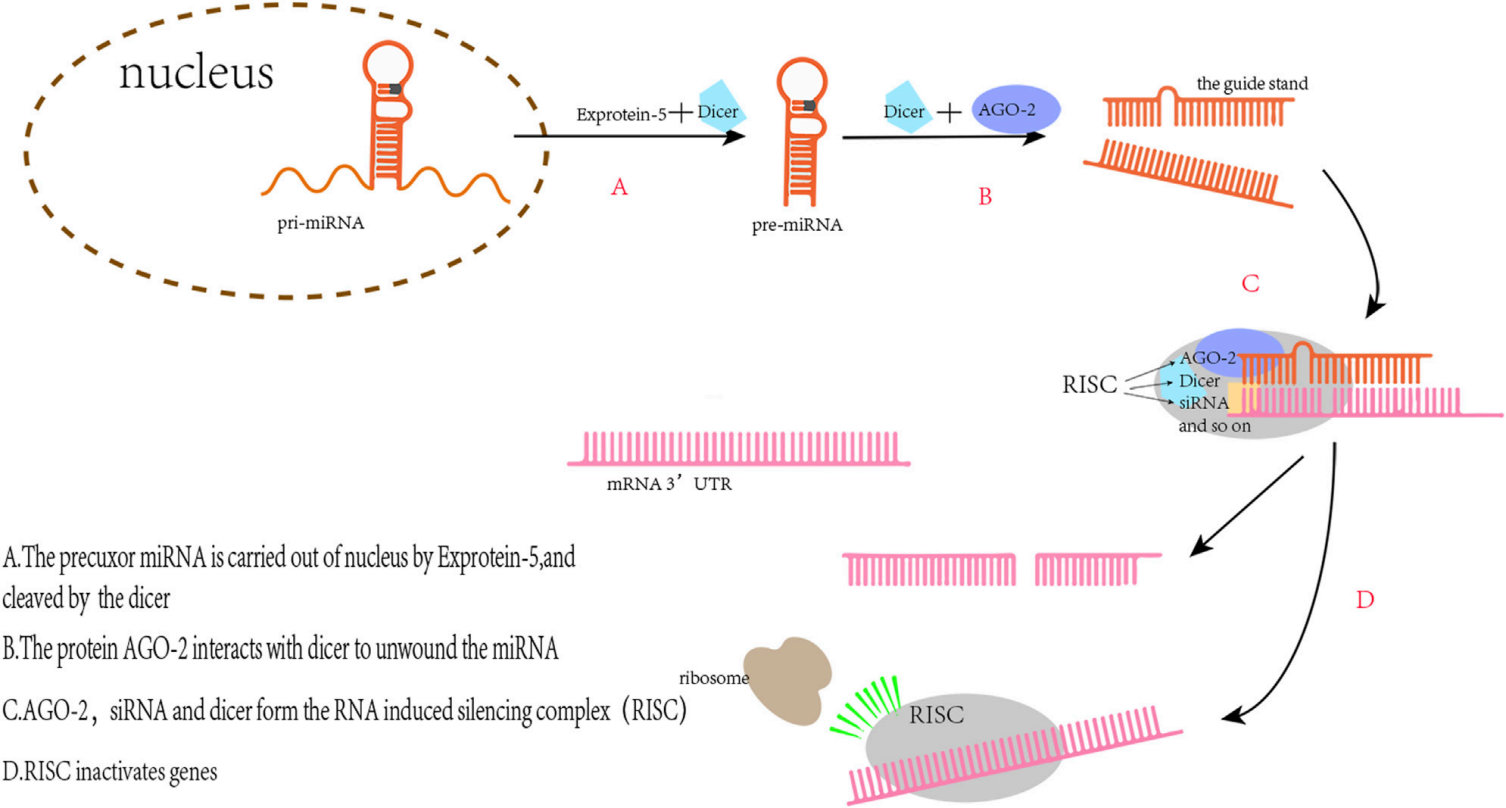
The principle of miRs regulating gene expression. The pre-miRNAs transported into the cytoplasm are complementary to Dicer to produce double-stranded RNA with a length of 22 nt. One of the two strands, the miRNA, binds to the RNA induced silencing complex (RISC) and then binding with the target mRNA to silence the mRNA expression.

MiRNAs are non-coding nucleotides of about 22 bases in length that regulate mRNA expression by binding to the 3′-untranslated region (3′UTR) of mRNA to affect various physiological processes *in vivo* (Figure 2) (Bartel, 2018). In recent years, miRNA levels *in vivo* have been thought to be linked with the diagnosis and prognosis of sepsis. For example, the level of serum miR-133a in mice with cecal ligation and puncture (CLP)-induced sepsis was significantly increased 6 h after modeling (Tacke et al., 2014); In lipopolysaccharide (LPS)-induced sepsis mice, elevated miR-199a expression in macrophages led to ARDS (Liu et al., 2018). This is due to miR-199a binding to the 3′UTR of silent information regulator 1 (SIRT1) mRNA inhibiting the anti-inflammatory effect of alveolar macrophages; MiR-107 induced tumor necrosis factor-α (TNF-α) secretion in endothelial cells by targeting dual specificity phosphatase 7 (DUSP7), which led to renal tubular cell injury during sepsis in mice and resulted in AKI (Wang et al., 2017). MiRNAs have also been associated with various cardiac diseases. For example, miR-122 can induce morphological and functional abnormalities of cardiomyocytes and increase myocardial apoptosis by acting on the Hand2/Drp1 mitochondrial cleavage pathway in mice with sepsis (Shi et al., 2021). Knockout of miR-21 in mice exacerbates cardiac hypertrophy in primary aldosterone syndrome, whereas overexpression of miR-21 confers a cardioprotection effect (Syed et al., 2018). These studies suggest that miRNAs play a key role in the pathogenesis, diagnosis, and treatment of sepsis and various cardiac diseases.

### 4.2 The role of miRNAs in the pathogenesis of SCM

#### 4.2.1 miRNAs regulate immunity in the SCM

**FIGURE 3:**
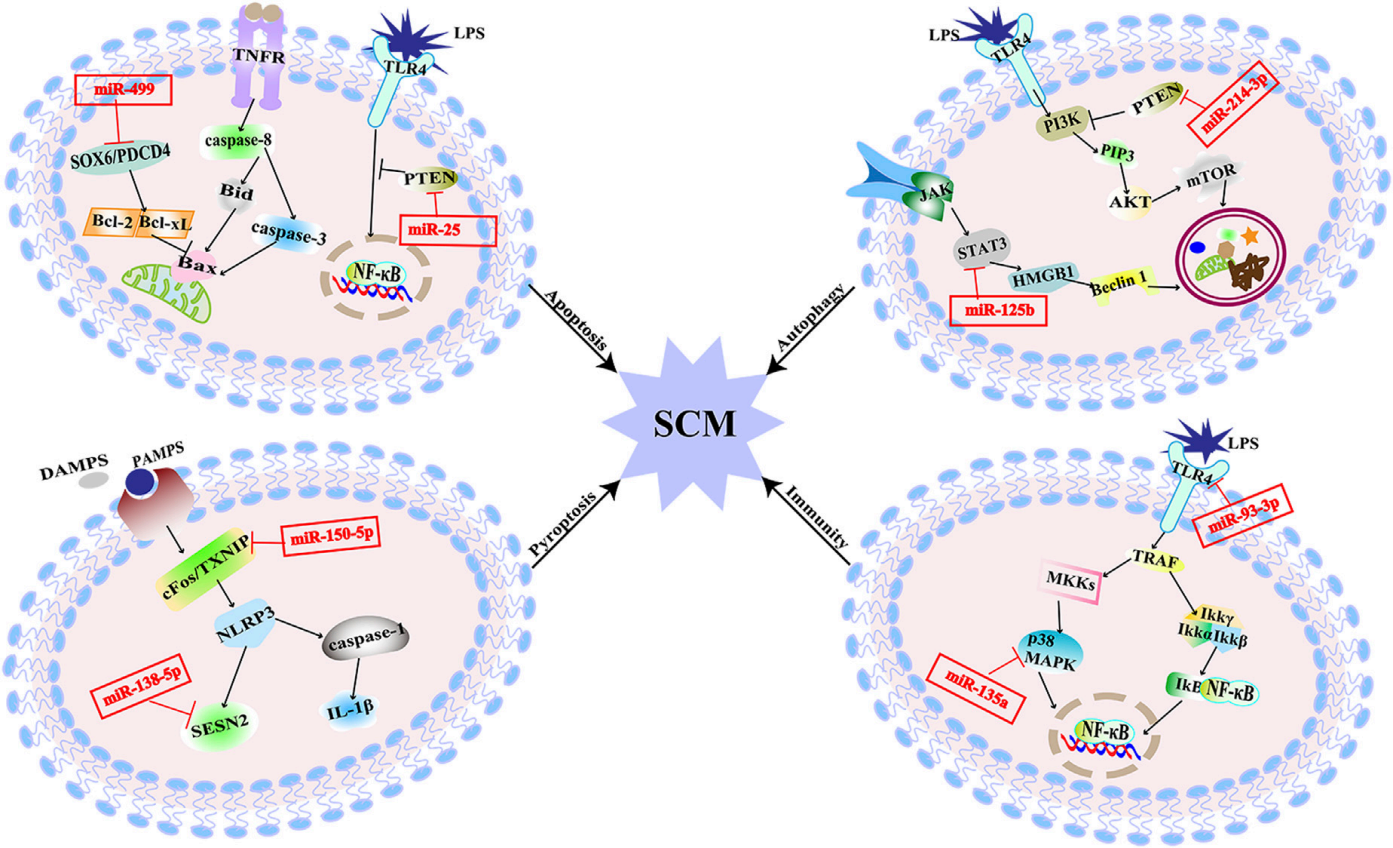
MiRNAs are involved in multiple pathogenesis of SCM. MiR-25 alleviates SCM by regulating TLR4/NF-κB signaling pathway to inhibit apoptosis. MiR-499 aggravates SCM by regulating SOX6/PDCD4 to over-activate apoptosis. MiR-125b inhibits autophagy and aggravates SCM through STAT3/HMGB1 signaling pathway. MiR-214-3p inhibits excessive autophagy by activating the PKB/mTOR pathway by silencing the PTEN gene to improve SCM. MiR-150-5p aggravated SCM through pyroptosis mediated by c-Fos/TXNIP axis. MiR-138-5p promotes pyroptosis and exacerbates SCM by inhibiting SESN2. MiR-93-3P promotes inflammation and aggravates SCM through TLR4/NF-κB/p65 axis; miR-135a promotes inflammation and exacerbates SCM through the P38-MAPK/NF-κB pathway.

There are literature indicating that balancing immune responses and reducing immune damage are of great significance for the prevention of SCM (Wang et al., 2021). Pathogens recognize corresponding receptors and start an immune response. Pattern recognition receptors include Toll-like receptors (TLRs), and NOD-like receptors (NLRs), which mediate excessive inflammatory mediators activated by corresponding signal pathways and cause damage to histiocytes (Kumar et al., 2011). MiRNAs target different sites of signal pathways to regulate the inflammation (Figure 3). The expression of miR-93-3p in H9c2 cells exposed to LPS is reduced, which increases the mRNA expression of TLR4. Increased expression of TLR4 mediates the activation of nuclear factors-κB/p65 (NF-κB/p65). The activation of the pathway initiates an inflammatory response in myocardial cells (Tang et al., 2018). Furthermore, upregulation of miR-135a levels in septic mice activates the P38 mitogen-activated protein kinase (P38-MAPK)/NF-κB pathway, the activation of this signaling pathway produces a large number of inflammatory factors such as interleukin-6 (IL-6), TNF-α, and interleukin-1β (IL-1β), which aggravates myocardial injury and cardiac dysfunction (Zheng et al., 2017). MiR-702-3P inhibits the expression of the oligomeric domain containing protein 1 (NOD1) gene by binding to the 3′-UTR region of the NOD1 mRNA of cell nucleotides. In LPS-treated septic H9c2 cells, the level of MiR-702-3P decreases, activating the NOD1 mediated signaling pathway, thereby exacerbating myocardial cell damage (Liu et al., 2022). The expressions of inflammatory factors such as TNF-α and IL-6 were increased by both of the two pathways. The increased TNF-α and IL-6 could inhibit cardiac function and further aggravate SCM (Kumar et al., 1996). To sum up, these miRNAs affect SCM by regulating the production of inflammatory factors downstream of the immune response signaling pathway.

As one of the most important cells of the innate immune system, macrophages play an important role in the immune response. MiRNAs may regulate cardiac function by affecting macrophages, which may be one of the important targets of SCM. LPS upregulates miR-146a expression in cardiomyocytes and macrophages. MiR-146a binds to the 3′UTR of the mRNA of tumor necrosis factor receptor-associated factor 6 (TRAF6) and interleukin-1 receptor-associated kinase 1 (IRAK1) to inhibit gene expression of TRAF6 and IRAK1, thereby negatively regulating the TLR/NF-kB pathway and alleviating sepsis-induced cardiac dysfunction in mice (Gao et al., 2015). Macrophage transfection with miR-146a altered macrophage inflammatory responses reducing inflammatory cell infiltration and inflammatory cytokine production. Studies have shown that the downregulation of miR-223 in SCM promotes TLR-triggered production of IL-6 and IL-1β in myeloid cells of mice, which leads to increased cytokine levels in circulation and results in defects in myocardial contractile function (Wang et al., 2014). A recent study in mice by Chen et al. showed that the downregulation of miR-223 triggers the production of IL-6 and IL-1β in macrophages (Chen et al., 2012). Thus, in SCM, downregulation of miR-223 most likely triggers IL-6 and IL-Ιβ production in macrophages, leading to cardiac dysfunction.

In summary, upregulation and downregulation of miRNAs can promote the occurrence of SCM by regulating the immune response.

#### 4.2.2 miRNAs regulate programmed cell death in the SCM

Apoptosis is a form of cell death showing as nucleus pyknosis, and karyorrhexis but maintenance of an intact plasma membrane, which maintains the cellular homeostasis. It can be initiated through the mitochondrial pathway associated with the B-cell lymphoma-2 (Bcl-2) family or the cell death receptor pathway involving activation of the tumor necrosis factor receptor (TNFR) family members (Chang et al., 2007). Cardiomyocyte apoptosis results in myocardial contractility impairment, which is associated with impaired cardiac function in sepsis (Smeding et al., 2012). The downregulation of miR-499 in LPS-stimulated H9c2 cells attenuates the inhibition of its downstream target genes, sex-determining region [SRY]-related HMG box (SOX6) and programmed cell death factor 4 (PDCD4), thereby over-activating myocardial mitochondria-mediated endogenous apoptosis. This process leads to the increased expression of Bcl-2 family proapoptotic proteins, exacerbating sepsis-mediated myocardial injury (Jia et al., 2016). MiR-361- 5p can inhibit the Wnt axis by targeting G protein-coupled receptor-4 (Lgr4) to promote myocardial apoptosis, and finally aggravate myocardial injury induced by LPS in septic mice (Li et al., 2022). X-linked inhibitor of apoptosis protein (XIAP) is one of the inhibitors of caspase in apoptosis (Sun et al., 2020). Decreased inhibition of miR-192-5p targeting the 3′UTR of XIAP mRNA in rats treated with LPS promotes apoptosis, thereby exacerbating SCM. The phosphatase and tensin homologue deleted on the chromosome 10 (PTEN) gene, as the target gene of miR-494-3p, is related to cell survival. In sepsis, the level of miR-494-3p in rat cardiomyocytes is reduced and facilitates the activation of PTEN-mediated cardiomyocyte apoptosis, resulting in excessive myocardial injury (Wu et al., 2019). Other miRNAs that directly target PTEN include miR-25. The decreased expression of miR-25 targets the PTEN and its downstream TLR4/NF-κB pathway to trigger myocardial apoptosis in septic rats (Yao et al., 2018). Besides that, it has been found that mitochondrial RNA damage can lead to mitochondrial dysfunction and eventually lead to apoptosis, which is also related to SCM (Yao et al., 2015). MiR-17-5p specifically binds to the 3′UTR of Poly (ADP-ribose) polymerase 1 (PARP1) mRNA, inhibits PARP1/human high mobility group box 1 (HMGB1) axis-mediated mitochondrial RNA damage, and then reduces cardiomyocyte apoptosis induced by mitochondrial damage in rats. Circular RNA TLK1 (CircTLK1) attenuates sepsis-induced cardiomyocyte apoptosis by reducing mitochondrial DNA damage through sponging miR-17-5p (Qiu et al., 2021). The above findings suggest that miRNAs could be involved in sepsis induced myocardial injury by regulating apoptosis (Figure 3).

Autophagy refers to a cellular degradative pathway that transfers damaged proteins and organelles to the lysosome in the form of autophagosomes, characterized by the accumulation of autophagic vesicles. Recent studies have found that autophagy might play a major role in SCM. In SCM, the proper level of autophagy is a protective response, but excessive autophagy can lead to cardiomyocyte damage, which is a double-edged sword (Yuan et al., 2009; Han et al., 2018). The signal transducer and activator of transcription 3 (STAT3) mediated HMGB1 signal pathway is hyperactivated during SCM, which leads to a hindrance of autophagic. The reduction of miR-125b may facilitate this process and exacerbate SCM by targeting STAT3 in rats (Yu et al., 2021). It has also been found that miR-22 can aggravate myocardial injury by acting on Sirt1 to inhibit autophagy in mice (Wang et al., 2021). MiR-214-3p can activate the protein kinase B/mammalian target of the rapamycin (PKB/mTOR) pathway by silencing the PTEN gene. That is presented as inhibiting excessive myocardial autophagy to improve cardiac function in septic mice (Sang et al., 2020).

Cell pyroptosis is a form of programmed cell death distinct from apoptosis, manifested by caspase-1-mediated rupture of the cell membrane and the release of mature inflammatory mediators, which is an essential part of the inflammatory response (Bergsbaken et al., 2009). The mature IL-1β produced during the process of pyroptosis can aggravate the inflammatory damage of SCM and further impair cardiac function (An et al., 2021). In LPS-induced SCM rats, miR-138-5p increased the expression of pyroptosis-related proteins by inhibition of Sestrin2 (SESN2). Reduced miR-150-5p levels in LPS-induced SCM rats promote c-Fos/thioredoxin interacting protein (TXNIP) axis-mediating pyroptosis. These account for an overwhelming cascade that leads to further myocardial injury (Wang et al., 2021).

### 4.3 miRNAs can be used as an indicator of SCM diagnosis

MiRNAs are not only involved in the pathogenesis of SCM but also can serve as diagnostic markers. The potential role of known miRNAs in the diagnosis of SCM is summarized in two ways. One is to use circulating miRNAs directly as diagnostic markers to diagnose SCM according to their upregulation or downregulation. This is becaus miRNAs can be present as stable molecules in plasma and other body fluids, and the level of miRNAs in serum samples is associated with cardiac function in SCM patients (Manetti et al., 2020). Some miRNAs are altered early in SCM patients, this character is advantaged in clinical detection (Benz et al., 2016). For example, miR-21-3p is elevated in the serum of SCM patients at levels proportional to NT-proBNP and cTnT, therefore it presumably can be used as a specific predictor of SCM (Wang et al., 2016). Some miRNAs that are significantly reduced in the serum of SCM patients, such as miR-495, which is downregulated in patients can be used as an early indicator for the diagnosis of SCM (Guo et al., 2019). The other is to examine the expression levels of genes targeted by miRNAs in the circulation. A transcription factors (TFs)-miRNA-target gene network was constructed by bioinformatics analysis of the differential expression of miRNAs between the SCM group and the control group (Chen et al., 2020). Several key miRNAs associated with SCM in mice such as the miR-29 family and the miR-30 family were identified. And key genes, such as CCL2, STAT3, MYC, and SERPINE1, are considered as potential biomarkers and therapeutic targets. These study populations are relatively small, and direct evidence of a functional role for miRNAs in SCM remains lacking. If miRNAs are used for clinical diagnosis, a larger population will be needed to verify the present results.

### 4.4 miRNAs may serve as precise therapeutic targets for SCM

#### 4.4.1 Regulation of expression of miRNA

Several animal experiments showed that some miRNAs were downregulated in septic mice, and the recovery or over-expression of miRNAs could reduce the process of myocardial inflammation and apoptosis. Chemical synthesis of miRNA agomir can restore or upregulate the content of miRNAs *in vivo*, thus alleviating myocardial injury. Infusion of miR-195-5p agomir into mice can reduce myocardial injury by targeting ATF6 to inhibit inflammation and apoptosis of cardiomyocytes. This experiment demonstrates that miR-195-5-p agomir can restore downregulated miR-195-5p to attenuate SCM (Xia et al., 2022). The miR-181b targets the HMGB1, which is an inflammatory mediator in late sepsis. MiR-181b agomir suppresses cardiomyocyte apoptosis and protects septic rats from myocardial injury (Ling et al., 2021). In addition to miRNAs with known targets of action, there are also miRNAs with unknown clear targets to improve SCM. MiR-940 was downregulated in CLP-treated rat models. When injected miR-940 agomir into rats, the concentrations of TNF-α and IL-6 in serum is reduced and myocardial cardiac function is improved. It is suggested that miR-940 agomir could alleviate SCM (Zhang et al., 2021b). The above miRNA replacement therapy is to restore or upregulate miRNAs *in vivo* by infusing chemically synthesized miRNA agomirs to alleviate myocardial injury.

The expression of some miRNAs is significantly increased in SCM mice, and antagonizing the above miRNAs can alleviate myocardial injury in mice. At present, anti-miRNAs therapy in animal experiments mainly relies on chemically synthesized antagomir. In mice with LPS-induced sepsis cardiac dysfunction, miR-21-3p antagomir directly targeted SH3 structural domain protein 2 (SORBS2) to reduce ultrastructural damage in mitochondria and improve myocardial contractility. And finally alleviates cardiac dysfunction (Wang et al., 2016). Injection of miR-451 antagomir into septic rats reduces the production of inflammatory factors and significantly alleviates myocardial injury. This study suggests that miR-451 antagomir attenuates myocardial inflammation induced by sepsis (Wang et al., 2020). After CLP induced myocardial injury, the expression of miR-328 was upregulated in rats. Injection of miR-328 antagomir could downregulate miR-328 and protect cardiac function (Sun et al., 2020).

In recent years, some miRNA inhibitors developed for stable expression in animal models have been referred to as “microRNA sponges" (Denzler et al., 2014). They inhibit miRNAs function via a competitive endogenous RNA (ceRNA) mechanism, leading to the upregulation of target gene expression (Zhang et al., 2022). Long non-coding RNA (LncRNAs) have been found to be miRNA sponges, which means, lncRNAs regulate target genes of miRNAs by binding to miRNAs, attenuating the original role of miRNAs on target genes (Thomson and Dinger, 2016).

MiR-93-5p can aggravate the inflammatory response by inhibiting SORBS2, a mechanism associated with SCM. lncRNA H19 promotes SORBS2 expression by binding to miR-93-5p, and attenuates LPS-induced apoptosis and inflammatory response in H9c2 cells (Shan et al., 2021). MiR-24 inhibits the expression of XIAP and promotes the protection of cardiomyocytes from inflammatory damage and oxidative stress. LncRNA CYTOR can inhibit the expression of miR-24 by binding to it, reducing myocardial cell damage during sepsis in mice, and ultimately achieving the goal of attenuating the injury of cardiomyocytes during sepsis (Chen et al., 2020).

#### 4.4.2 miRNAs improve SCM with the help of carriers

Due to the body’s protection mechanisms and the properties of miRNAs, naked miRNAs cannot pass through the phospholipid bilayer *in vivo*, and may even be eliminated by cells (Dowdy, 2017). MiRNA agomirs or antagomirs rely on systemic injections for their effect *in vivo*, which is expensive and less effective (Mellis and Caporali, 2018). Therefore, finding suitable carrier delivery vehicles for miRNAs is crucial for treating diseases, as it helps miRNAs reach their targets stably *in vivo* (Sarkar and Kumar, 2021). Currently, established miRNA delivery vehicles include lentiviral vectors and exosomes (Rupaimoole and Slack, 2017).

Infusion of miR-23b lentivirus expression system into the aortic root of CLP-induced cardiomyopathy mice through micro-catheter inhibited NF-κB-mediated inflammatory responses. Upregulated miR-23b decreases caspase-3 activity and apoptosis rate by inhibiting TLR-mediated NF-κB to attenuate myocardial injury (Cao et al., 2019). Upregulation of miR-146a expression by injection of miR-146a lentiviral vector into CLP mice attenuates NF-κB-mediated inflammation and thus reduces cardiac dysfunction in septic mice (Gao et al., 2015).

Mesenchymal stem cells (MSCs) “help” transport miRNAs mainly through their secreted phospholipid vesicles called “exosomes” (Lai et al., 2010). Exosomes are luminal vesicles secreted by eukaryotic cells, which contain a variety of substances, such as proteins, mRNAs, and miRNAs. Depending on specific signals, exosomes arrive at target cells to release carried substances. The exosomes are considered to be a cell-independent mode of communication that can reach target cells and release the substances they carry based on specific signals (Lakkaraju and Rodriguez-Boulan, 2008). Lentiviral vectors may induce host immune response, exosomes as endogenous vesicles can avoid this risk. Exosomal delivery of miR-223 into cardiomyocytes of SCM mice improves survival by inhibiting inflammation-related genes Sema3A and STAT3 (Wang et al., 2015).

In addition to mesenchymal cells, exosomes can also be derived from macrophages. M2 macrophages secreted exosomes containing large amounts of miR-24-3p, injection of these into mice with LPS-induced sepsis can attenuate myocardial pathological damage. This is because miR-24-3p inhibits tumor necrosis factor superfamily member 10 (Tnfsf10), which exerts anti-inflammatory effects (Sun et al., 2022).

### 4.5 Possible adverse effects after using miRNA as a treatment option

The most serious adverse effect is that a miRNA usually targets dozens or even hundreds of genes, making it difficult to achieve specific gene silencing, which leads to a series of unpredictable consequences. This characteristic of miRNA has recently been termed “too many targets for miRNA effect” (TMTME) referring to one miRNA may regulate the expression of lots of genes without clear effect (Zhang et al., 2021a). There have been few news reports that miRNA drugs are in complete clinical trial progression, mostly attributed to the above feature described. Secondly, the transfected miRNAs may have competing effects with endogenous miRNAs on mRNA sites, which in turn increases the expression of their targeted genes, making it difficult to achieve the desired effects (Khan et al., 2009). Finally, Due to the instability of miRNAs, delivery of miRNAs requires chemical modification to avoid rapid degradation *in vivo*, which may impair their biological activity (Corey, 2007; Traber and Yu, 2023).

## 5 Conclusion

As described in Figure 3, it has been revealed that miRNAs participate in SCM through multiple pathways, and the known signaling pathways are intertwined to form a network. Compared with the existing symptomatic treatments, targeting a specific miRNA may be more effective. And due to the conserved nature of miRNA sequences, the feasibility from basic experiments to clinical practice is relatively large. The clinical prospect of miRNAs against SCM is promising.

## 6 Outlook

For the clinical application of miRNAs for SCM, some problems still need to be solved. For example, it is necessary to clarify the precise signaling pathways involved in the pathogenesis of the disease so that the precise therapeutic targets or therapeutic capabilities can be determined. In addition, as for miRNA-mediated autophagy which is a “double-edged sword” mechanism, it is necessary to precisely control the expression of miRNAs to achieve therapeutic purpose.
